# What's in the Box? Punishment and Insanity in the Canadian Jury Deliberation Room

**DOI:** 10.3389/fpsyg.2021.689128

**Published:** 2021-06-30

**Authors:** Susan Yamamoto, Evelyn M. Maeder

**Affiliations:** ^1^Department of Psychology, Carleton University, Ottawa, ON, Canada; ^2^Institute of Criminology and Criminal Justice, Carleton University, Ottawa, ON, Canada

**Keywords:** insanity, juror decision-making, punishment, moral foundations theory, not criminally responsible on account of mental disorder

## Abstract

In insanity cases, although the defendant's eventual punishment is legally irrelevant to the jury's decision, it may be psychologically relevant. In this three-part mixed-methods study, Canadian jury eligible participants (*N* = 83) read a fictional murder case involving an insanity claim, then took part in 45-min deliberations. Findings showed that mock jurors who were generally favourable towards punishment had a lower frequency of utterances that supported the Defence's case. A qualitative description of keyword flagged utterances also demonstrated that mock jurors relied on moral intuitions about authority, harm, and fairness in justifying their positions. These findings may have application in crafting effective Judge's instructions and lawyer's opening statements.

“*Our collective conscience does not allow punishment where it cannot impose blame.”*

—Judge David Bazelon

## Introduction

The Canadian legal system takes a clear stance on the issue of criminal culpability and mental disorder^1^. According to Section 16 of the Criminal Code: “No person is criminally responsible for an act committed…while suffering from a mental disorder that rendered the person incapable of appreciating the nature and quality of the act or omission, or of knowing it was wrong” (Criminal Code of Canada, 1985). Hence a person may be found Not Criminally Responsible on Account of Mental Disorder (NCRMD) if the party raising the issue can prove it is more likely than not that, during the crime, the defendant had a mental disorder that precluded a guilty mind. Rather than traditional punishment via the criminal justice system, a successful NCRMD claim will result in psychiatric care or in some cases release. This provision is in tension with a longstanding culture of hostility towards the insanity defence in Canada (Maeder et al., 2015) and the United States (Hans, 1986). Every person has the constitutional guarantee to a fair trial by an impartial tribunal (Canadian Charter of Rights and Freedoms, 1982, Section 11d). A pressing issue is therefore whether the legal system lacks adequate safeguards to combat juror partiality.

Despite the rarity of NCRMD (i.e., up to 6.08 per 1,000 among decisions averaged over 5 years in three provinces; Crocker et al., 2015b) and the strict review process, the public tends to see it as a frequently exploited loophole that sets dangerous offenders free (Skeem et al., 2004). Unfortunately, this misconception can result in mock jurors' inability to correctly apply this law

^1^

when appropriate (Bloechl et al., 2007; Maeder et al., 2015). Researchers have remarked on the kinship between insanity defence attitudes and different punishment orientations (Skeem et al., 2004; Breheney et al., 2007). However, existing proposed remedial measures do not target all punitive motives equally. For example, reassuring jurors about deterrence only targets utility focused punishment, but this negative bias might also be seated in desires for retribution. It is well-established that insanity myths (e.g., that the defence is commonly used) play a key role in aversion towards the defence (Skeem et al., 2004), but it is possible that certain moral intuitions interfere after correcting misconceptions.

Considering the many issues that NCRMD defendants potentially face, it is clear that we must probe whether negativity towards legal insanity reflects a lack of information on the part of the public or moral intolerance of the defence. It is also necessary to glimpse inside the jury deliberation process to fully diagnose the problem. In doing so, we can examine not just opinions about NCRMD, but also how they fare in response to persuasion. Whereas, researchers have made gains in understanding individual juror decisions in insanity cases, only one (U.S.) study of which we are aware (Wheatman and Shaffer, 2001) has examined group deliberation in insanity cases. In a three-part study, we used mixed methodology to examine whether endorsement of retributive (“an eye for an eye”) and utilitarian (“for the greater good”) principles to support punishing vs. avoiding punishment related to mock juror decisions as well as what types of themes emerged in jury deliberation. Of note, section 649 of the Canadian Criminal Code prohibits jurors from discussing court proceedings post-trial, and so it is necessary to employ simulation studies.

## Insanity

Insanity is a legal rather than psychiatric term. The law defines a mental disorder as follows for the jury: “any illness, disorder, or abnormal condition that impairs the human mind and its functioning” (National Judicial Institute, 2014). A defendant is always presumed to be innocent until the Crown (prosecution) has proven beyond a reasonable doubt that he or she is guilty. Mental disorder is an exception; the party raising the NCRMD claim must prove that it is “more likely than not” that the defendant had a mental disorder at the time of the crime. The standard of proof is lower than beyond a reasonable doubt, which is articulated in model instructions.

Data from the National Trajectory Project on Individuals Found Not Criminally Responsible on Account of Mental Disorder in Canada (NTP, Crocker et al., 2015a) showed that 70.9% of primary diagnoses at the index NCRMD verdict were psychotic spectrum disorders (e.g., schizophrenia) while 23.2% were mood disorders. Consequently, in this paper we focus on juror decision-making in an NCRMD case involving schizophrenia. Persons with schizophrenia seem to be subject to a high degree of stigma, wherein laypeople are sceptical of the potential for treatability and associate the illness with dangerousness (Angermeyer and Dietrich, 2006; Day et al., 2007). Therefore, this group might be especially vulnerable to insanity defence bias.

There are at least two dimensions that comprise negative insanity related attitudes: one concerning myths about the defence, and one relaying notions of strict liability (i.e., “If you do the crime, you do the time,” Skeem et al., 2004). Hence some laypersons are simply recalcitrant on the matter of mental disorder and criminal responsibility. Members of the public tend to erroneously believe that defendants who are found NCRMD are released into the community without provisions. Those who estimate that insanity defendants immediately go free are less likely to support the insanity defence and are more likely to vote guilty (Skeem et al., 2004). Some researchers have proposed the use of focused education to combat bias against defendants who are reasonably using the insanity defence. Indeed, Hans (1986) showed that those with higher levels of education in general are more likely to support the insanity defence. Similarly, Maeder and Laub (2012) found that psycho-legal education (i.e., an undergraduate class that featured lectures on the insanity defence) improved student attitudes towards the defence. In two studies, Maeder et al. (2015) attempted to educate Canadian mock jurors about the NCRMD defence in hopes that it would improve relevant attitudes. In the first study, education produced the predicted difference in NCRMD attitudes, but it did not affect verdict decisions. In contrast, the second study revealed no such effect of focused education on attitudes, or verdict decisions. Hence, education is not always an effective remedy to negative insanity defence attitudes.

## Punishment Orientation

Punishment can be defined as a “a negative sanction intentionally applied to someone perceived to have violated a law, rule, norm, or expectation” (Vidmar and Miller, 1980, p. 568). People tend to rely on two main types of arguments when punishing others: retributivism and utilitarianism. Retributivism, which follows from Kant's (1785/Kant) Deontology, holds that punishment must be proportionate to the wrongdoing (Schedler, 1980). Unlike retributivism, the cornerstone of utilitarianism—commonly associated with Bentham (1789/Bentham), and later championed by Mill (1859/2008)—is consequentialism. An act must maximise the aggregate good for those affected by it. Therefore, incapacitation, rehabilitation, and specific/general deterrence are utilitarian punishment practises. While a retributivist is not *per se* opposed to these goals, they are likely to prefer balancing the scales of justice. Laypersons do not always appreciate the distinction between these viewpoints (Carlsmith et al., 2002).

Recent work illustrates the possibility that some people are generally punishment motivated, while others focus on the risks associated with punitive acts. Yamamoto and Maeder (2019) sought to rectify the apparent difficulties in operationally defining punishment orientation by creating four scales that measured which retributive and utilitarian principles work in tandem vs. in tension. This work was based on studies showing that more logically calculated decisions tend to require suppression of an automatic aversion to doing harm (Valdesolo and DeSteno, 2006; Greene, 2009). However, research indicates that some people do not seem to experience this aversion (Bartels and Pizzaro, 2011). Hence, Yamamoto and Maeder (2019) theorised that people would differ in terms of seeing punishment as itself rewarding. We termed people on the punishment-prone end of the spectrum as having a “permissive” punishment orientation (i.e., will permit punishment across a number of contexts) and those on the punishment-averse end of the spectrum as having “prohibitive” punishment orientation (i.e., tend towards prohibiting punishment where possible).

By virtue of the fact that jurors are assessing the defendant's control over the act, labelling insanity as a “guilt” decision is somewhat of a misnomer. Rather, jurors will dictate whether the defendant is to be incarcerated or treated, which is at least in part a question of punishment beliefs. The legal system intends for jurors to only rely on evidence of whether the defendant had a mental disorder at all (and whether it precluded a guilty mind), but this is not to say that jurors will avoid retrospective justifications based on punishment goals. If the mere fact of an indictable offence triggers a punitive need (that must be sated), then jurors may match the incoming information to the least dissonant storey. The concept of strict liability (i.e., “you do the crime, you do the time”; Skeem et al., 2004) deals directly with the outcome of insanity trials. Endorsing such a viewpoint implies that prison is preferred over institutionalisation.

## Overview

Researchers have made great gains in understanding individual juror decision-making in insanity cases, but the jury deliberation process remains a black box. NCRMD decisions are a natural moral conflict. Jurors are presented with an incident of harm, and yet told that it is not necessarily punishable. Hence there is likely a third unspoken question inherent to jurors' assessments: whether NCRMD satisfies the goals of punishment. In Study Parts 1 and 2, we tested whether participant scores across the permissive and prohibitive punishment dimensions predicted verdict decisions and deliberation content. In Study Part 3, we observed how jurors attempted to persuade each other of their positions.

## Study Part 1

Jurors have notoriously negative attitudes towards the insanity defence, and yet correcting misinformation alone appears insufficient to change verdict decisions (Maeder et al., 2015). It is possible that considering jurors' punishment orientation will help to tease apart different motivations for this negativity. In Study Part 1, we analysed relationships among individual differences and pre-deliberation verdicts.

### Hypotheses

Following Yamamoto and Maeder (2019), we predicted that permissive retributivism and utilitarianism would be associated with increased likelihood of a guilty verdict. We also expected that prohibitive retributivism and utilitarianism would be associated with decreased likelihood of a guilty verdict and more favourable insanity defence attitudes.

### Method

#### Participants

Overall, 172 people interacted with the online survey. Of those, 107 completed Phase 1 (i.e., 65 people did not complete the survey). A total of 24 participants dropped out of the study prior to Phase 2. Remaining participants were 83 (47 men, 34 women, 2 transgender individuals) Canadian jury-eligible community members (i.e., citizens at least 18 years of age with no indictable offences) recruited online via Kijiji (a classified ads website similar to Craigslist), having a mean age of 29 (*SD* = 11.5) and ranging from 18 to 62. The majority of participants (63.9%) identified as White, while 18.1% identified as Black/African-Canadian, 4.8% as Middle Eastern, 2.4% as Aboriginal Canadian/Native Canadian/First Nations, 2.4% as East Indian, 1.2% as Asian, 1.2% as Hispanic/Latino, and 6% as another group.

##### A Priori Sample Size

A power analysis for a two-tailed Pearson or Point-biserial correlation using G^*^Power yielded a minimum sample size of 82 for a medium effect size (0.30) at α = 0.05, with 0.80 power. However, due to the exploratory, mixed methods nature of the study, the main rationale for the sample size overall rested on the concept of saturation. Saturation roughly constitutes reaching the point at which new information has been exhausted; that is, there are sufficient data to be trustworthy (Fusch and Ness, 2015). Underscoring the lack of established rules for achieving saturation, Francis et al. (2010) outlined four principles that might justify these decisions in theory-driven content analysis. In the first two steps, researchers must *a priori* select an initial analysis sample and a stopping criterion. The initial analysis sample should be based on the minimum sample size needed to satisfy stratification factors (e.g., diversity of age, ethnicity). The stopping criterion dictates the number of additional interviews after new ideas are considered exhausted. For the current studies, due to inherent limitations in recruitment numbers, we first considered a jury sufficient if there were at least five members (half of the minimum permitted attrition to continue a trial). Notably, we also collected data from multiple juries with the minimum Canadian legal requirement of 10 people. Given the large size of the groups, we used six juries as our initial sample size and collected from four extra juries as our stopping criterion. Indeed, these criteria yielded a gender balanced sample, with age diversity, with somewhat greater racial diversity as compared to the racial composition of Canada.

#### Materials: Phase 1

##### Insanity Defence Attitudes

Participants completed the 19 items of the Insanity Defence Attitudes-Revised Scale (IDA-R; Skeem et al., 2004) adapted to a Canadian context, which comprises two latent factors (injustice and danger, strict liability). The Strict Liability scale pertains to the extent that a person believes that mental disorder is irrelevant to criminal responsibility (e.g., “I believe that people should be held responsible for their actions no matter what their mental condition”) and showed strong internal consistency (α = 0.85). The Injustice and Danger scale pertains to fears about misuse of the defence and the potential threat to public safety (e.g., “As a last resort, defence attorneys will encourage their clients to act strangely and lie through their teeth to appear mentally ill”) and showed strong internal consistency (α = 0.86). Participants rated their agreement on a 7-point Likert-type scale ranging from 1 (*strongly disagree*) to *7* (*strongly agree*).

##### Punishment Orientation

Participants also completed the 17 items of the Punishment Orientation Questionnaire (POQ; Yamamoto and Maeder, 2019), which comprises four scales that measure the principles people rely on when thinking about appropriate punishment in the criminal justice system. The Prohibitive Utilitarian scale measures the extent to which participants believe punishment should be goal-oriented and benefit society (e.g., “Punishment should be about looking forward to improve society, not backward to address the criminal's misdeeds”); the scale showed strong internal consistency (α = 0.82). The Permissive Utilitarian scale measures the extent to which participants are willing to give strict punishment to the aim of deterrence (e.g., “an overly harsh punishment may be necessary to prevent others from committing the same crime”); the scale showed strong internal consistency (α = 0.82). The Prohibitive Retributive scale captures aversion to the risks of punishment (e.g., “It is better to let 10 guilty criminals go free than to punish 1 innocent person”); the scale showed strong internal consistency (α = 0.81). Finally, the Permissive Retributive scale captures blame of the criminal label and desires for retribution (“Criminals are bad people and get what is coming to them”); the scale showed strong internal consistency (α = 0.84). Participants rated their agreement on a 5-point Likert scale ranging from 1 (*strongly disagree*) to 5 (*strongly agree*).

##### Demographics Survey

Participants completed a demographics survey, which included race, gender, occupation, and level of education. They were also asked to provide their religious and political affiliations (if any), and whether they personally know someone with a mental disorder. Finally, participants were asked to indicate where their political beliefs fell on a sliding liberal to conservative scale.

#### Materials: Phase 2

##### Trial Transcript

Participants heard one page of model jury instructions adapted from the National Judicial Institute (2014) about the essential elements of the charge and requirements for NCRMD, as well as the burden of proof. We created an ~8-page trial transcript loosely based on Clark (2006), which describes a second-degree murder charge against a man who stabbed his roommate. Of note, the defence does not dispute that the accused committed the physical act, but that he did not have the requisite guilty mind. The transcript begins with opening statements from the Crown and Defence. The Crown alleges that the accused is a violent man who snapped in response to a heated argument. The police officer who arrested the defendant serves as a Crown witness and provides evidence that the defendant was attempting to flee with the victim's wallet. The Defence alleges that the accused had paranoid schizophrenia at the time of the crime and specifically had Capgras delusion. A psychiatrist (whose gender was left ambiguous) testifies to this effect. The psychiatrist describes the diagnostic criteria for the defendant's disorder and gives an explanation for the lack of clear history of mental disorder. The trial ends with closing statements from the Crown followed by the Defence. Each participant filled out an individual verdict form as well as a verdict confidence rating, ranging from 0 (*not at all confident*) to 10 (*very confident*) after reading the trial transcript. Participants selected from guilty, not guilty, or not criminally responsible on account of mental disorder. For all analyses reported, NCRMD was coded as 0 and guilty was coded as 1. Mock jurors were not permitted to take notes.

##### Pre-deliberation Instructions

Participants heard two pages of instructions about the criteria for NCRMD and how to decide whether the defence meets those requirements. These instructions also reiterate special rules on the burden of proof and reasonable doubt. Finally, participants were instructed on logistics of the deliberation (e.g., selecting a foreperson, unanimity requirements; National Judicial Institute, 2014). The foreperson was instructed to complete a verdict form, selecting from guilty, not guilty, NCRMD, or unable to reach a verdict.

##### Procedure: Phase 1

Three to 4 days prior to the deliberation, participants followed a link from a recruitment notice on Kijiji. After passing juror eligibility screening, participants selected the appropriate time slot for the coming Saturday (and were told to check back the following week for alternative sessions). They were then directed to the Phase 1 informed consent form, followed by the (counterbalanced) IDA-R/POQ and lastly the demographics survey. After being directed to a new survey, participants either entered an email address and received further instructions for the deliberation phase, or they withdrew from the study.

##### Procedure: Phase 2

Participants were seated in order of arrival (i.e., the first to arrive was assigned as Juror #1, which was displayed on the table and above the seat). Once participants completed informed consent, the research assistant read the pre-trial instructions (about 5 min) and handed out the trial transcripts with individual verdict forms, for which participants were given about 15 min.

After all materials were collected, the research assistant read the pre-deliberation instructions and provided the jury verdict form. Sommers (2006) used a limit of 60 min and found that on average deliberations in a sexual assault case ranged from ~38–50 min. Given practical limitations to coding time and funds, participants were told that the deliberation would last no longer than 45 min; a clock was displayed on a screen. The research assistant then left the room for the duration of the deliberation and waited in a smaller lab office next door to observe participants through a two-way mirror and audio-visual system. Participants could wave at the two-way mirror any time for assistance or once they reached a verdict. The research assistant was instructed not to provide any further information about the case, except to re-read passages of the instructions if questioned. Once the deliberation finished, participants completed the post-deliberation questionnaire. They were then debriefed and compensated with $40 for their time.

## Study Part 1: Results and Discussion

### Attitudinal Variables

First, we assessed bivariate relationships among the attitudinal variables^2^ (see Table 1). In line with expectations, the POQ dimensions showed moderate relationships with the IDA-R dimensions. It was conceivable that strict liability would be more strongly related to retributivism, and injustice and danger more strongly related to utilitarianism (Skeem et al., 2004; Maeder et al., 2015). However, permissive utilitarianism and permissive retributivism showed moderate positive relationships with both dimensions. Prohibitive retributivism showed a weak negative relationship with both IDA-R dimensions. Prohibitive utilitarianism showed a weak negative relationship with strict liability only, having no significant linear association with injustice and danger. Those higher in the tendency to focus on the positive societal impact of punishment were less likely to believe that mental disorder is irrelevant to a crime, but no less likely to believe the insanity defence is misused or threatens public safety. Additionally, political orientation was significantly related to permissive retributivism, *r* (76) = 0.29, *p* = 0.01, such that higher identification with conservatism was associated with greater permissive retributivism, or greater identification with liberalism was associated with lower permissive retributivism.

**Table 1:** Bivariate relationships among attitudinal measures.

	**1**	**2**	**3**	**4**	**5**	**6**
1. Strict Liability	1					
2. Injustice & Danger	0.49^**^	1				
3. Permissive Retributive	0.46^**^	0.42^**^	1			
4. Permissive Utilitarian	0.36^**^	0.41^**^	0.70^**^	1		
5. Prohibitive Retributive	−0.28^*^	−0.31^*^	−0.30^*^	−0.39^**^	1	
6. Prohibitive Utilitarian	−0.36^**^	−0.12	−0.45^**^	−0.22^*^	0.24^*^	1

** *p ≤ 0.001.*

* *p ≤ 0.05*.

### Criterion Variables

Next, we assessed bivariate relationships between the attitudinal measures and pre-deliberation (i.e., individual) dichotomous verdict decision; Table 2 displays these relationships. A continuous verdict variable (the multiplicative product of guilty vs. NCRMD and verdict confidence) was also included, ranging from −10 (very *confident in an NCRMD verdict*) to 10 (*very confident in a guilty verdict*). The confidence measure contained a “0” option to essentially allow for jurors to be undecided between guilty and NCRMD. The majority of jurors individually rendered a guilty verdict (*n* = 50, 61%) prior to deliberation, whereas 32 (39%) chose NCRMD. Only one participant voted not guilty, and so this case was dropped from analyses. Individual *dichotomous* verdict decision (where NCRMD was coded as 0 and guilty was coded as 1) related only to the dimensions of the IDA-R. Permissive retributivism and utilitarianism shared a significant positive relationship with *continuous* verdict, such that those higher on the traits were more confident in a guilty verdict, and those lower on the traits were more confident in an NCRMD verdict. One possible explanation for this finding is that people with stronger convictions about their verdict decision were more likely to have strongly developed beliefs about punishment.

**Table 2:** Bivariate relationships between attitudinal measures and outcome variables.

	**Individual dichotomous verdict**	**Individual continuous verdict**
Strict Liability	0.23^*^	0.28^*^
Injustice & Danger	0.25^*^	0.32^*^
Permissive Retributive	0.24	0.30^*^
Permissive Utilitarian	0.16	0.22^*^
Prohibitive Retributive	0.00	0.00
Prohibitive Utilitarian	−0.08	−0.12

*N = 82.*

* *p ≤ 0.05*.

There are some notable limitations to Study 1. Most significantly, the case appears to be somewhat guilt leaning, which may itself contribute to the relationship with punishment orientation or insanity defence attitudes. The POQ is a relatively new measure, and so it is unclear to what extent permissive and prohibitive retributivism and utilitarianism are context dependent. For instance, it could be that particularly heinous cases render permissive retributive concerns more central. However, Yamamoto and Maeder (2019) reported that presenting the POQ before vs. after a death penalty case did not significantly influence findings, suggesting that the measure could be context resistant. The deliberation analyses help to underscore relevant idiosyncrasies of the trial transcript by showing what narratives participants created from the evidence. In brief, it appears punishment orientation indeed seems to play some role in mock jurors' beliefs about the insanity defence. The next step was to ascertain what topics mock jurors actually leveraged in attempts to publicly defend their positions.

## Study Part 2: Overview

Study Part 2 concerned the examination of transcriptions of mock jury deliberation sessions. Hsieh and Shannon (2005) described the method of *directed content analysis* as a means to further investigate established phenomena. In Part 2 we employed a deductive method, given that previous findings on the insanity defence guided a priori creation of a coding manual.

### Hypotheses

#### Hypothesis 1a: Crown Position-Taking

Researchers have theorised that some jurors dislike the insanity defence because it implies the defendant is not punishable (Skeem et al., 2004; Breheney et al., 2007). Consequently, we predicted that more permissive punishment orientation would be associated with a higher frequency of Crown position-taking. This code was applied when participants expressed the opinion that the defendant was likely guilty (e.g., “It's obvious this guy was guilty,” “Yes, he is responsible”).

#### Hypothesis 1b: Defence Position-Taking

We predicted that lower permissive punishment orientation would be associated with a higher frequency of Defence position-taking. This code was applied when participants expressed the opinion that either prong of NCRMD might be met, or that the defendant was NCRMD (e.g., “he's obviously mentally ill,” “he didn't know what he was doing”).

#### Hypothesis 2: Defendant Disposition

We predicted that more permissive punishment orientation would be associated with a higher frequency of references to the defendant's ultimate disposition (i.e., what would happen to the defendant following trial).

### Coding

Two independent coders were trained to assess each individual utterance for the categories listed in the codebook, which contained principles arising from Skeem et al.'s (2004) IDA-R and Yamamoto and Maeder's (2019) POQ. Coders also looked for references to Crown/Defence evidence/arguments and evaluations (e.g., “the Defence's case was weak”) as well as explicit votes (including non-verbal hand-raises). These codes were generated based on the trial transcript as well as a single pilot student deliberation. We used Cohen's kappa as a metric of reliability and resolved disagreements through discussion. Following Sommers (2006), two coders assessed 20% of the juries, and one coder assessed the remaining juries. Notably, some codes were used infrequently, which yielded perfect agreement when they were not present, but did little to illustrate coders' ability to detect that content.

Roughly mirroring Greene et al. (2008), coders examined uninterrupted utterances on a single topic (i.e., “idea units,” p. 208), with sentences as the rough grain size (Chi, 1997). Implications for this choice of granularity are explored in the General Discussion section. Because people speak less formally in comparison to written communication, punctuation was only one potential marker for a coding unit; the utterance had to express a coherent thought. These units ranged from two-word ideas (e.g., “I agree”) to several word run-on sentences (e.g., “And I think he knew that like it was uh it was yea, y'know, yea like, I i-illegal yes, but morally –”). Coders were conservative in applying labels to passages. A slight degree of ambiguity resulted in a label of “other”; for example, it might have been unclear with what concept a participant was agreeing. Codes were only applied where there was substantive content. The scheme was exhaustive for each unit (i.e., only one code was applied to each sentence). Table 3 provides a breakdown of the major topics.

**Table 3:** Rate of major topics (per total utterance count).

	**Crown**		**Defence**		**Disposition**		**Mental disorder**		**Legal instructions**	
**Group**	**Count**	**Proportion**	**Count**	**Proportion**	**Count**	**Proportion**	**Count**	**Proportion**	**Count**	**Proportion**
Jury 1	22	0.04	17	0.03	10	0.02	46	0.09	68	0.13
Jury 2	21	0.06	2	0.01	5	0.02	15	0.05	13	0.04
Jury 3	15	0.02	15	0.02	14	0.02	63	0.10	48	0.08
Jury 4	24	0.06	5	0.01	11	0.03	37	0.10	15	0.04
Jury 5	3	0.01	24	0.04	21	0.04	25	0.04	80	0.14
Jury 6	14	0.02	20	0.03	56	0.09	22	0.03	44	0.07
Jury 7	16	0.02	12	0.02	12	0.02	37	0.05	63	0.09
Jury 8	17	0.04	11	0.03	24	0.06	45	0.12	39	0.10
Jury 9	17	0.04	9	0.02	28	0.06	38	0.08	47	0.10
Jury 10	7	0.23	0	0.00	0	0.00	0	0.00	3	0.10

Overall, the range of Kappas demonstrated fair reliability^3^ (0.54–1.00, Cohen, 1960; see Supplementary Material). Several values were at the lower end of the conventionally acceptable Kappa range. Low base rates can decrease Kappa even in cases of high agreement (Xu and Lorber, 2014) and so again the infrequency of some codes warrants caution in interpreting these values. Tables 4, 5 display an overall summary of the features of each jury (group features and demographics, respectively).

**Table 4:** Group characteristics broken down by discussion group.

**Group**	**Outcome**	**Proportion guilty verdicts**	**Size**	Straw poll timing^*^	**Time (Minutes)**
Jury 1 (A)	Hung	0.56	9	326 (549)	41.00
Jury 2 (B)	Guilty	0.67	6	325 (327)	28.00
Jury 3 (C)	Hung	0.33	9	Informal Sequential (618)	47.00
Jury 4 (D)	Guilty	0.82	11	1 (372)	28.00
Jury 5 (E)	NCRMD	0.25	8	Informal Sequential^**^ (557)	42.00
Jury 6 (F)	NCRMD	0.50	6	Formal sequential (637)	36.00
Jury 7 (G)	Hung	0.50	6	391 (730)	32.00
Jury 8 (H)	Hung	0.75	12	14 (383)	47.00
Jury 9 (I)	Hung	0.67	9	29 (451)	43.00
Jury 10 (J)	Guilty	0.86	7	26 (31)	1.72

*“Informal sequential” indicates that jurors provided a verdict along with a rationale and/or informally expressed positions in turn*.

*“Formal sequential” indicates that jurors confirmed a position one after another*.

* *Utterance number at which a formal poll was initiated and completed, followed by total number of utterances in brackets*.

** *Poll vetoed by a juror*.

**Table 5:** Demographics broken down by discussion group.

**Group**	**Age**		**Gender**		**Racial composition**		**Know person with mental disorder**	
						
	**Mean (SD)**	**Man**	**Woman**	**Trans**	**White**	**Another race**	**Yes**	**No**
Jury 1 (A)^*^	30.2 (9.7)	6 (66.7%)	3 (33.3%)	0	6 (66.7%)	3 (33.3%)	4 (44.4%)	5 (55.6%)
Jury 2 (B)^**^	25.0 (5.1)	5 (83.3%)	1 (16.7%)	0	2 (33.3%)	4 (66.7%)	4 (66.7%)	2 (33.3%)
Jury 3 (C)^*^	40.4 (16.8)	4 (44.4%)	5 (55.6%)	0	7 (77.8%)	2 (22.2%)	5 (55.6%)	4 (44.4%)
Jury 4 (D)^**^	26.6 (9.4)	7 (63.6%)	3 (27.3%)	1 (9.1%)	5 (45.5%)	6 (54.5%)	7 (63.5%)	4 (36.4%)
Jury 5 (E)^***^	24.8 (9.1)	6 (75.0%)	2 (25.0%)	0	4 (50.0%)	4 (50.0%)	5 (62.5%)	3 (37.5%)
Jury 6 (F)^***^	30.0 (14.0)	3 (50.0%)	3 (50.0%)	0	5 (83.3%)	1 (16.7%)	4 (66.7%)	2 (33.3%)
Jury 7 (G)^*^	23.8 (7.2)	4 (66.7%)	2 (33.3%)	0	3 (50.5%)	3 (50.5%)	4 (66.7%)	2 (33.3%)
Jury 8 (H)^*^	22.7 (4.6)	5 (41.7%)	7 (58.3%)	0	9 (75.5%)	3 (24.5%)	7 (58.3%)	5 (41.7%)
Jury 9 (I)^*^	26.9 (8.1)	3 (33.3%)	5 (55.6%)	1 (11.1%)	7 (77.8%)	2 (22.2%)	9 (100%)	0 (0%)
Jury 10 (J)^**^	40.0 (12.7)	4 (57.1%)	3 (42.9%)	0	5 (71.4%)	2 (28.6%)	4 (57.1%)	3 (42.9%)

* *Hung,*

** *Guilty,*

*** *NCRMD*.

## Study Part 2: Results and Discussion

For each hypothesis, we tested a separate hierarchical linear model using HLM Software 7 (Raudenbush et al., 2011). Given that the dependent variables were based on counts rather than continuous measures and had several zero counts (i.e., were positively skewed, but not suitable for regular transformations), the data did not meet the assumptions of ordinary linear regression (Gardner et al., 1995). We therefore specified a Poisson distribution with over-dispersion (Raudenbush et al., 2011). Further, because jurors varied in number of utterances, total individual utterance count was included as an exposure variable, which accounted for different “chances” for observation of each code category. First, we examined the null model to ascertain whether the jury that a participant was in significantly contributed to the variance in the dependent variable (i.e., ran a model without any independent variables). Second, we added grand-mean-centred punishment orientation score^4^ (where higher scores denote more permissive orientation) as a level 1 predictor and executed a random-intercepts only model. Results reported represent population average models.

### Hypothesis 1a: Crown Position-Taking

In Hypothesis 1a, we predicted that more permissive punishment orientation would be associated with a higher frequency of Crown position-taking (i.e., expressing the opinion that the defendant was likely guilty). Using Crown position-taking frequency as the dependent variable, the null model was significant, χ^2^(9) = 26.87, *p* = 0.002, demonstrating that level 2 grouping significantly contributed to the variation in Crown position-taking frequency. Punishment orientation did not significantly predict Crown position-taking (*B* = 0.38, *SE* = 0.20, *p* = 0.060).

### Hypothesis 1b: Defence Position-Taking

In Hypothesis 1b, we predicted that lower permissive punishment orientation would be associated with higher frequency of Defence position-taking (i.e., expressing the opinion that either prong of NCRMD might be met, or that the defendant was NCRMD). Using Defence position-taking frequency as the dependent variable, the null model was non-significant, χ^2^(9) = 9.60, *p* = 0.384, demonstrating that level 2 grouping did not significantly contribute to the variation in Defence position-taking frequency. There was a significant effect of punishment orientation on Defence position-taking frequency, *B* = −0.56, *SE* = 0.17, *p* = 0.002, such that those with a higher permissive orientation had a lower frequency of Defence position-taking utterances.

### Hypothesis 2: Disposition

In Hypothesis 2, we predicted that more permissive punishment orientation would be associated with a higher frequency of references to the defendant's ultimate disposition (i.e., what would happen to the defendant following trial). Using disposition-related utterance frequency as the dependent variable, the null model was significant, χ^2^(9) = 60.56, *p* < 0.001. The effect of punishment orientation on disposition-related utterances was non-significant, *B* = 0.21, *SE* = 0.16, *p* = 0.192.

### Implications

Results of three hierarchical linear models revealed that punishment orientation predicted a higher frequency of utterances that explicitly supported the Defence, but not those supporting the Crown or discussing the Defendant's eventual punishment. These findings support previous work on punishment and the insanity defence. In their study of judge's instructions and jury deliberations, Wheatman and Shaffer (2001) found that while including information about the defendant's disposition did not influence individual verdicts, juries who heard these instructions were significantly more likely to find the defendant NGRI. Thus, consideration of the defendant's disposition can elicit leniency. It is perhaps unsurprising, then, that in the current study, punishment attitudes related to leniency (i.e., supporting the Defence) rather than harshness. Because the burden is on the Defence to prove insanity, jurors who are punishment-motivated might look the same as those who simply have a high threshold of proof that the accused had a mental disorder at the time of the crime. Although the burden to prove the accused had a mental disorder is a lower standard than beyond a reasonable doubt (i.e., “more likely than not”), mock jurors may not have made this distinction. In essence, some jurors might have been able to override an aversion to traditional punishment in favour of following the law, while others might have simply been punishment motivated. However, those who are willing to make inferences from the evidence that support an insanity narrative may be particularly averse to traditional punishment. Individuals with greater strength of conviction with respect to prohibitive punishment orientation might also have needed to defend their position intensely given the burden of proof.

### Limitations

Results of Studies 1 and 2 provided support for the role of punishment orientation in informing both jurors' initial survey of the evidence as well as their public advocacy for those positions. However, there are a handful of limitations to consider. First, the data are underpowered to examine group outcomes. Because the primary interest in this study was descriptive rather than inferential, we did not examine relationships between variables of interest and final verdict outcomes. Second, many of the categories from the initial code bank were simplistic; it is unclear from the quantitative content analysis alone how participants engaged the idea of punishment. The central role of narratives in juror decision-making arguably beckons qualitative methods. If the idea is that jurors co-construct meaning in a unique discursive context, then quantitative methods become limiting. Accordingly, we conducted a third exploratory study that relied on inductive methods, to the aim of situating the data in participants' narratives and usage of punishment orientations.

## Study Part 3: Overview

Haidt (2001) has maintained that evaluative feelings about another's actions defy easy articulation and emerge automatically without consciously weighing rational premises. One salient example is the common aversion to a romantic relationship between siblings (Haidt, 2001). People seem to have a sense that it is wrong even if they cannot justify that feeling, a phenomenon known as “moral dumbfounding.” As Haidt (2001) described, people tend to maintain their initial position on an issue, often with awareness at their inability to produce reasonable counterarguments. A key tenet of this social intuitionist account is the assertion that moral “reasoning” is retroactive (Saltzstein and Kasachkoff, 2004). Similarly, as Breheney et al. (2007) remarked, jurors might have a general aversion to the notion that those found insane are not punishable in the traditional sense, regardless of lacking a guilty mind.

Haidt and Graham (2007) proposed that there are five moral intuitions (also called foundations): fairness/reciprocity, harm/care, authority/respect, sanctity/purity, and ingroup/loyalty. Each foundation may be rejected or accepted as a basis for moral virtues (i.e., qualities that make a person “good”). The fairness dimension concerns notions of equality and equal protection. For instance, people tend to be concerned with the justness of the procedures used to make decisions, sometimes more so than with the actual outcome (Tyler, 1984). The harm dimension concerns preference for actions that promote safety rather than suffering. For instance, some may find it virtuous to prevent the suffering of the greatest number of people, while others cannot tolerate harm to a single individual (Foot, 1967). The authority dimension concerns deference to hierarchy. For instance, some cultures may emphasise subordination whereas others value challenges to authority (Haidt and Graham, 2007). The sanctity dimension extends more primitive notions of uncleanliness and danger to the moral realm. For example, religious virtues may dictate appropriate bodily activities such as sex (Haidt and Graham, 2007). Finally, the ingroup dimension concerns the natural tendency to socially categorise others and preference loyalty to those perceived as similar. Moreover, moral foundations share a connection with the primary factors thought to influence jurors' consideration of evidence (Devine, 2012). For instance, the authority foundation might speak to mock jurors' assessment of the credibility of the witnesses and other evidence. Other mock jurors might be preoccupied with the moral violation itself (i.e., direct harm done to another) or the fairness of not receiving traditional punishment.

While the general goal of content analysis is to reduce a large amount of data to a more concise rendering of a phenomenon, as Hsieh and Shannon (2005) summarised, it is also a tool for subjective interpretation. Qualitative content analysis moves further on the interpretive spectrum as compared to Quantitative content analysis (Sandelowski, 2000). However, Qualitative Description can be considered relatively “low-inference,” in contrast to other Qualitative methods such as grounded theory (Sandelowski, 2000, p. 335). In the summative content analysis approach, keyword and content searches serve to identify language that is manifestly representative of a construct. Then, moving beyond manifest content, the researcher tries to understand the context in which those terms are mobilised, in attempts to uncover alternative meanings (Hsieh and Shannon, 2005). Hence, this method fills a gap by providing a more nuanced picture of the role that the variables of interest play in persuasion. The purpose of Study 3 was to provide a closer look at the general content identified in Study 2 and to explore usage of language related to moral intuitions (i.e., moral foundations theory, Graham et al., 2009). Taking direction from Hsieh and Shannon (2005), Erlingsson and Brysiewicz (2017), and Sandelowski (2000), we first completed a qualitative description of utterances whose content was about the defendant's disposition.

We used the Linguistic Inquiry Word Count (LIWC, Pennebaker et al., 2015) program to flag utterances containing language related to the moral intuitions of authority, fairness, and harm^5^. The moral foundations dictionary, which comprises a collection of words associated with each foundation, has been extensively contextually validated (Graham et al., 2009), and so it was a more reliable gauge of some of the constructs of interest as compared to the initial codes. Graham et al. (2009), with the assistance of a team of researchers, created this dictionary by searching for words theoretically associated with each dimension and then examining use of those words in transcribed religious sermons. The dictionaries for fairness and harm served as a proxy for retributive desires. The authority dictionary served to further probe mock jurors' discussions about the psychiatrist and about the law itself.

## Study Part 3: Method

We conducted a summative content analysis, in which we coded utterances relating to a-priori content of interest. After using LIWC (Pennebaker et al., 2015) to conduct a keyword search for moral foundations language, we content analysed all resulting passages. Following Erlingsson and Brysiewicz (2017), the first author first read and re-read the utterances from all 10 juries, to get a general overview of the content. Next, utterances were broken down into meaning units, in a similar fashion to Greene et al. (2008). Meaning units were defined as an utterance by a single juror on a single topic, which had independently substantive meaning. Meaning units were then condensed into smaller representative phrases, still staying as close as possible to the participants' words. Codes were then assigned to each utterance, which captured their general essences. These codes along with exemplar utterances were combined into a code manual, and then conceptually similar codes were placed together. Finally, categories were assigned that captured the relationship between these conceptually similar codes. Given that qualitative content analysis moves beyond manifest content to encompass some subjective interpretation, drawing parallels to quantitative methods of quality assurance (e.g., inter-rater reliability) is a complicated endeavour. Mayring (2014) thus recommends that a second researcher serve as quality control by “supervising” and “checking” the first coder's work (undertaken by the second author). Because we stayed relatively towards the descriptive end of the spectrum, codes should reasonably read as present or absent.

## Study Part 3: Results and Discussion

### Disposition

This analysis probed the question of how mock jurors engaged ideas about the defendant's potential punishment. Five general categories emerged with respect to what would happen to the defendant after the trial: the effectiveness of prisons, the conditions of prisons, the jury's duty in considering punishment, desires for rehabilitation, and desires for incapacitation. Hence, the majority of discussion surrounded utility-based concerns, although there were a handful of references to ideas of just deserts (e.g., “scot-free”).

First, several mock jurors indicated that prisons are not rehabilitative, which tended to accompany discussions about the amount of suffering in prisons. One NCRMD voter indicated that a defendant should prefer jail over institutionalisation, citing that periods of institutionalisation are typically longer than prison sentences in these cases. There were also varying beliefs about the conditions of corrections centres. In the following exchange, one NCRMD voter (J12H) argues to guilty voters that prison facilities have poor conditions (i.e., are “not a great place to be”).

J5H: Also like, being in jail, is not the worst thing that can happen to you.
J10H: (Inaudible) medication.
J5H: Yea, actually, if they get him help, in jail, then.
J12H: Our prison facilities here are (inaudible). You have to share toenail clippers with forty other guys, it's not a great place to be. But he will have to take his medication.
J7H: But I don't think you can, I don't think you can judge like: “Oh, it's gonna be really hard in jail.” Like I can't bring that into, like, my decision as to whether or not he is. I'm sorry.

The above passage also illustrates a third category, which pertained to the jury's duty in considering the defendant's disposition. Some jurors urged others to “follow the law.” Others acknowledged that the defendant's disposition was not legally at issue, but nonetheless discussed the topic.

J5I: And, I mean there is also, I mean, the, um, prison could also be, a, danger to, um, somebody, with, a, mental illness, it can go both ways, but I can't, I'm not sure.
J6I: But a murderer is dangerous to a mental facility [laughs].
J4I: (Inaudible).
J5I: I'm not sure, it's appropriate for us to consider sentencing?
[Talking over each other].
J5I: There's a whole, there's a whole thing of, I-I mean, I'm personally, strongly, of the opinion that the prison system needs reform, but like, I still, I mean.
[Talking over each other].
J1I: She did, specifically.
J5I: Yea, so I-I, unfortunately I don't think, we can, we're allowed to, like, consider that, I think we just have to go off of the facts (inaudible) different stage with different deliberations.

Some jurors espoused the idea that it was not their duty to consider punishment or treatment.

J1C: The one thing that's guaranteed though if you're sent to the psychiatric facility, he will be treated. Okay? You send him to jail, he's gonna do his time, he's gonna get out, and he'll be back in fruitbat land.
J9C: Yeah, but we're not, we're not debating that.
J2C: Yeah, that's not the issue that (inaudible) to me, it whether not whether*[sic]* or not it's effective, I know it's not effective. It's whether or not this specific individual, if (inaudible) to receive the treatment, they would receive because they have a mental illness or whether they should be put to the prison system because they have all their faculties. Well, I agree with you the prison system does nothing to rehabilitate criminals, but to me that's not the issue here.

This passage also highlights a fifth category pertaining to jurors' desires for incapacitation and protection of society. Relatedly, the timing of the defendant's release was a point of contention. Some juries settled on the idea that the defendant would not be released until safe.

J6E: Do you guys know that hospital, like how does that work?
J8E: I think you stay there until the professionals deem you fit, or get you under control.
J6E: And then right back into normal clothes?
J8E: I think so. And then, I think, definitely probably back to a psychiatrist.
J8E: But it's not just like you spend a week and.
J6E: Yeah, it would be a while.

Other jurors maintained a concern that the defendant was a danger to society dependent on their decision. This belief persisted among those who questioned the defendant's motive in avoiding prison. For instance, two jurors who previously expressed concerns that the lawyer coached the defendant had the following exchange:

J2A: So what happens next time?
J4A: Does he kill again? Because he… he's [scare quotes] schizophrenic? Are you guys good with your decision? [Gestures at J3 and J6].

A fifth category pertained to jurors' desires for the defendant's rehabilitation. It was commonplace for mock jurors to either indicate that treatment was needed or to use questions about treatment as a persuasive tactic, which were sometimes successful. One juror attempted to persuade the eventual lone holdout (J1E) of eight:

J3E: If he were to have paranoid schizophrenia, do you believe that jail is the right thing for him?
J1E: No.
J3E: No, you don't.
J1E: The hospital.
J3E: Right.
J1E: If he was cured somewhere like that.
J3E: So, if I've not misunderstood, if is found guilty he would go to jail, if he was found not criminally responsible and he has a mental illness, he would be going to mental hospital or seeing doctors on a regular basis, is that right?
J1E: I would rather him have a (inaudible).
J3E: But isn't that what Not Criminally Responsible means?

The holdout juror changed positions by the end of the deliberation: “And I do like Not Criminally Responsible. The fact that he would be taken care of.”

Notably, there were a handful of utterances indicating an unfavourable attitude towards the language of NCRMD because it implies that the defendant is not punishable (e.g., J4F: “I just don't like how they phrased it…It makes it sound like he's not responsible like he's going to get away like”). There were also notions that NCRMD is a legal loophole (e.g., J4A: “And I also believe sometimes that Defence attorneys will tell that to their clients, umm, ya know?”). These ideas are reminiscent of strict liability and injustice and danger attitudes towards the insanity defence. In brief, mock jurors predominantly espoused concerns about rehabilitation, the conditions in and effectiveness of corrections, incapacitation, and their duty in considering punishment.

### Moral Foundations

This analysis addressed the question of how different moral foundations were rejected or accepted in persuading other jurors. The search for words related to the purity and ingroup foundations returned insufficient data for analysis. Therefore, we examined how jurors used language relating to the moral foundations of authority, fairness, and harm in justifying their positions. Of note, LIWC (Pennebaker et al., 2015) separately flags words with positive and negative valences, to give a sense of whether words constituted the foundations as “vices” or “virtues.”

#### Authority

LIWC (Pennebaker et al., 2015) yielded 6 utterances containing authority vice language and 64 containing authority virtue language. Both NCRMD and guilty voters leveraged principles of authority acceptance and rejection in their utterances but differed in terms of whose authority should be trusted. NCRMD voters deferred to the authority of the psychiatrist, as demonstrated in the following exchange on a hung jury, in which the eventual lone holdout (J1I) defended his position:

J1I: I think under the rules we were given we're engaging in a lot of speculation about what's, not in there, again I'm, I'm trusting the psychiatrists' diagnosis, and we're engaging…, In, what, to me, is a lot of tenuous speculation “well maybe the psychiatrist is wrong, maybe there's this, maybe there's that”—I think you guys are –[Talking over each other].
J1I: – insinuating a lot of the things, into, the case.
J5I: I do think it's an obligation of jurors, though, to consider the quality of the evidence, and my opinion was that I mean the psychiatrist doesn't, lack quality, but the quality of the evidence would be vastly improved by even, again, just one corroborating witness. And they didn't have that, and that makes me feel like that I, really want to believe them, but I just feel like I can't.

In the above passage, while Juror 1I insists that the authority of the psychiatrist be accepted, Juror 5I rejects the idea in favour of consideration of the Crown evidence. Similarly, one juror objected to reliance on the psychiatrist's authority: “J6F: to me it's weird cuz like we're just kinda like putting a label on this thing and saying it's like ok by like certain authorities uh… things are excusable because of this like black box that we don't know—or that I don't know anything about.” Rather than the authority of the psychiatrist, guilty voters tended to defer to the authority of the law. For instance, one juror who maintained a guilty verdict throughout deliberation persisted in the idea that the defendant did not have a diagnosis of schizophrenia: “Under the law, or under the hospital act of Canada, he is not certifiable schizophrenic.” However, given that this assertion is not factual, the participant appears to have been using law as an authority without any knowledge thereof. It was also common for guilty voters to rely on the phrase “that's the law.” This notion tended to accompany discussions about the defendant's punishment.

In terms of alternative usages that did not evince rejection or acceptance of this moral foundation, LIWC (Pennebaker et al., 2015) also flagged utterances related to the role of the Defence lawyer. For instance, jurors debated whether the Defence lawyer would encourage a client to erroneously plead NCRMD: “And then the thing, the case, for th-the lawyer, I mean he can't try to convince someone to like act crazy or lie as the lawyer you know…that gets you disbarred or get into a whole lot of trouble too.” Usages also encompassed jurors' attempts to establish their own credibility. Jurors who voted guilty sometimes tried to establish their own authority by indicating they had experience with law or forensics. Like guilty voters, NCRMD voters sometimes established their own credibility, but more often through experience working with persons with mental disorders. For instance, one juror repeatedly espoused facts about anti-psychotics. Specifically, they suggested that the defendant's anti-psychotic appeared to be working, and that this was evidence of a true mental disorder. NCRMD voters also sometimes downplayed their own experience in deference to others' expertise: “I mean, I'm not the doctor so.”

#### Fairness

LIWC (Pennebaker et al., 2015) yielded 39 utterances containing fairness vice language and 11 containing fairness virtue language. One of the most frequently flagged words related to the fairness moral foundation was “bias,” which appeared to encompass acceptance of this moral foundation in determining correct action. Mock jurors seemed to converge on the idea that bias was an important consideration but differed on whether it was present. NCRMD voters tended to cite the psychiatrist's professional reputation and neutrality. Guilty voters sometimes communicated the idea that the psychiatrist might be biased, ranging from the potential for anyone to be biased to outright dishonesty. Both NCRMD and guilty voters also called attention to their own potential for bias.

J6D: Well, I guess the bias is like, for me is I hang out with a lot of (inaudible) sick faces (inaudible). For me, like, I deal with a lot of kids where they are treated, they're like (inaudible) they're treated like (inaudible).
J9D: I don't know, I think just focus on that particular question. I mean, you can use all of your prior knowledge or your past experience, of course, but I think that based on what we have and then, that particular question, you have to decide for yourself. [turns to J10] What about you?
J10D: Yeah, I'm, um, again, I'm totally biased as well, but I'm—you know, based on what we have here, I'm definitely gonna say guilty. Unfortunately.

LIWC (Pennebaker et al., 2015) also captured concessions (e.g., “that's fair”), as well as parroting of legal language. In particular, the term “reasonable” is in the fairness dictionary and was primarily used in discussing the concept of reasonable doubt. Occasionally, jurors indicated that another's position was “reasonable.” Finally, “justice” and “justification” tended to appear in discussions of whether it was permissible for one to kill an alien or another person in self-defence. In sum, the keyword search for fairness flagged acceptances of this moral foundation, but not rejections of it.

#### Harm

LIWC (Pennebaker et al., 2015) yielded 195 utterances containing harm vice language and 37 containing harm virtue language. The software flagged every instance of the word “kill,” given that it represents a harm against another. Kill-related utterances had roughly three forms. First, both NCRMD and guilty voters occasionally acknowledged the harm done (e.g., “he killed a man”). Second, jurors debated the characteristics of “violent people,” for example: “I mean most of them either abuse animals before, or get in fights and assaults before the murder, they don't just…” Third, mock jurors focused on the victim's manner of death. For instance, they attempted to make inferences from the number of stab wounds, which were positioned as indicative of the defendant's emotional state:

J1F: I was—I was gonna say that one of the things I considered was how the victim was killed, in other words was it a… you know, a shot to the head, was it a single stab wound, but multiple stab wounds to the neck –
J5F: You're either angry or you're scared.
J4F: Well if you're angry you just [inaudible−9:43].
[All at once—inaudible].
J1F: It's like—stab, stab, stab, I don't know, I—I just felt that –
J3F: Yeah, I hear what you're saying.

Unsurprisingly, guilty and NCRMD voters diverged on who the defendant believed the victim was—an alien or his friend—and by extension, his motive for killing. Several jurors questioned the necessity/excessiveness of killing (i.e., that there were alternative choices). Relatedly, the keyword “protect” emerged in two distinct contexts. First, NCRMD voters tended to favour the narrative that the defendant believed it necessary to kill in order to protect society. Conversely, among guilty voters, the term “protect” arose more so in relation to the need to protect society from further violence. One juror even made explicit reference to the Punishment Orientation Questionnaire:

J4I: [Raises hand] Oh! OK! Right! The reason why I was saying that we should consider that is that, I feel like, if we're unsure, if we are leaning toward one way but we're really unsure, then we should go with the decision that benefits society, like. The questionnaire that we filled out? Online?
J5I: Ohhhh!
J4I: Is it that, if someone's responsible, is it that they get what they deserve? Or is it that, it's what, benefits society most, so I'm just saying if it's to the point where we're really indecisive? Then we should go with the decision which, is the most beneficial, or protecting of, people, including, inmates, but, I, just [raises hands] like.

As with the fairness dimension, LIWC (Pennebaker et al., 2015) flagged legal language, specifically several occurrences of “suffering from a mental disorder.” The term “suffering” appears in the National Judicial Institute model instructions. Understandably, the law appears to encompass multiple moral foundations.

### Implications

The intention for Study Part 3 was to provide a more complete understanding of the general constructs highlighted in Study Part 2. Following a qualitative description paradigm, we conducted a summative content analysis by examining utterances containing key words relating to moral foundations (Graham et al., 2009) and utterances related to the defendant's potential disposition. The data were presented to compare and contrast NCRMD and guilty voters' interpretations of case facts as well as their use of the same values in justifying positions.

As Graham et al. (2009) argued, the form of one's moral persuasions can be more interesting than the simple content. In general, results imply that jurors from different positions relied on similar rhetorical strategies, including logical if/then statements as well as appeals to intuition (e.g., “it just seemed like”). They seem to diverge on the premises presented in supporting those arguments; use of moral foundations related language helped to uncover what values might have motivated those interpretations. In a post outlining how lawyers can incorporate moral foundations language into their closing and opening statements, one litigation consultant remarked: “Without a theme, your case is just information: facts, claims, exhibits, instructions, and witnesses” (Broda-Bahm, 2015). This advice appears to be well-placed, given that moral foundations might feature in discussion.

In terms of discussion surrounding the defendant's eventual disposition, jurors' utterances encompassed several of the major goals identified by both psychologists and philosophers. However, the majority of these goals pertained to utilitarian concerns of rehabilitation, incapacitation, and deterrence. This finding is in line with research showing that when justifying decisions, people tend to prefer utilitarian reasons to retributive ones (Carlsmith et al., 2002). However, their preoccupation with the poor vs. acceptable conditions of prisons shares a kinship with retributive concerns (i.e., the amount of harm the defendant might experience). Passages flagged by the moral foundations dictionary provided some insight into jurors' more retributive driven arguments. Specifically, the harm dimension underscored concerns with the degree of damage done by the defendant (as well as future dangers). Findings seem to suggest that jurors do engage moral intuitions during deliberation. Moreover, appearance of this language provides further evidence of contextual validity for portions of Graham's et al. (2009) Moral Foundations Dictionary. Indeed, moral foundations related language is embedded into the law itself, which participants often echoed.

## General Discussion

This three-part study explored the role of punishment orientation in mock jurors' pre-deliberation decisions and deliberation content in response to a fabricated NCRMD case involving paranoid schizophrenia. Study Part 1 involved analysis of individual juror decision-making. Study Part 2 involved a directed content analysis and hierarchical linear models testing the relationship between punishment orientation and deliberation content. Finally, Study Part 3 more closely examined that content through a combination of keyword searches and qualitative description.

The Introduction opened with a quotation from Judge David Bazelon: “Our collective conscience does not allow punishment where it cannot impose blame.” Are jurors likely to agree? Our primary interest was what principles and strategies mock jurors relied on in attempting to defend their positions. The rationalist camp might say that punishment should indeed be a calculation based on a defendant's intentionality and of potential future consequences. Conversely, from an intuitionist perspective, one could say that the need for punishment alone imposes blame. Under a retributive framework, for example, justifications for punishment are retrospective; retribution is desired simply by virtue of a harm done. As Tebbit (2005) articulated, retributive oriented people punish because they want to.

The data can shed light on the relative emphasis certain jurors place on each strategy. In Part 3, we found that mock juror utterances occasionally featured virtually no reasoned or narrative justification of their perspective. Rather, they presented conclusions as self-evident. Mock jurors also evinced acceptance of three moral foundations, in line with Graham et al. (2009). These results lend support to an intuitionist account. On the other hand, jurors also seem to use reasoned arguments surrounding the burden of proof and lack of evidence. For example, several jurors articulated affectively based reasons for wanting to vote NCRMD (e.g., sympathy), but cited legal instructions as prohibiting reliance on those feelings, which could be interpreted as a cognitive override of intuitions. Overall, given that punishment orientation shared a significant relationship with juror decisions and utterance frequencies, it is at least clear that jurors do not discount punishment intuitions.

### Implications

It may be left to lawyers and psychiatrists to persuade jurors on the appropriateness of NCRMD. Results of content analyses from Parts 2 and 3 underscore at least three possible strategies. First, mock jurors appear concerned about whether they are permitted or ought to consider the defendant's ultimate disposition. In fact, these studies undermine the very notion that jurors are not in the business of deciding punishments. If jurors truly make decisions on the basis of intuitions (Haidt, 2001), then it is difficult to conclude that punishment is irrelevant to the decision. Therefore, perhaps instructions should be amended to explicitly broach what role punishment might play in their decision. Second, lawyers, through opening/closing statements or expert testimony might consider explicitly depicting either incarceration as rehabilitating or institutionalisation as punishing. Third, legal practitioners might consider the role of authority in juror decisions. Study Part 3 indicated that some NCRMD and guilty voters diverged on deference to the law vs. to the psychiatrist. Again, legal instructions explicitly permitting jurors to consider punishment might change decisions. Even where there is no opposing expert testimony, a second corroborating expert might be needed. In either case, a strategy might be to feature moral foundations language in opening statements. However, it is worth noting that even in the face of these changes, mock jurors could experience dumbfounding (Haidt, 2001).

### Limitations

There are a number of general limitations that require consideration. Chief among those limitations is the exploratory nature of the study, which resulted in a small-scale investigation lacking experimental manipulations. Although this restricted focus was intended to produce a rich rather than comprehensive project, several variables of interest were left unexplored. For instance, research underscores mental disorder stigma as a likely source of juror bias, which can vary as a function of mental disorder type (Yamamoto et al., 2017). Likewise, jurors' prototypes about mental disorders seem to influence decision-making (Skeem and Golding, 2001). These studies also cannot account for the intersectionality of experiences with mental disorder (Crenshaw, 1989). Defendant and participant characteristics (e.g., race, gender) tend to change jurors' perceptions. Additionally, notions of free will play a significant role in psychiatric vs. legal conceptualizations of the insanity defence (Rychlak and Rychlak, 1990). It is also likely that beliefs about free will are associated with prohibitive vs. permissive punishment orientations, given that the latter is marked by greater blame. Future researchers may therefore wish to measure general beliefs about human agency. It also bears mentioning that there are many other origins of beliefs about insanity, ranging from friends to the media and politicians; researchers should consider such sources of persuasion.

We did not control for group level influences as previous researchers have done, such as simultaneous vs. sequential voting (Davis et al., 1988), leaving the power of normative vs. informational influences ambiguous. The models in Part 2 were more simplistic than other possible models. It would be useful in future research to treat utterances as nested within jurors and to add time as a variable. Accounting for the presence or absence of certain topics later in deliberation might give us a sense of whether juries were evidence or verdict driven. Similarly, a time variable could provide information about whether more moralised issues drive deliberation or occur towards the end, after thorough discussion of evidence.

Although the qualitative analyses reported in this paper were largely descriptive in nature, “immaculate perception” is not possible (Beer as cited in Sandelowski, 2000, p. 335). It is necessary to acknowledge that all qualitative analyses feature some number of interpretive liberties. For instance, we chose to present findings by comparing and contrasting guilty and NCRMD voters, which might have influenced results. As such, the analysis does not focus on jurors who were relatively uncertain, but rather captures those who had stronger moral convictions. However, that interpretation more closely approximates our research question, and so we leave ideas about how uncertainty manifests in terms of swing votes to future researchers.

While the inevitable interpretability of the data can be an issue, by the same token, further interpretation would likely yield a richer picture. Again, our descriptive qualitative analysis does not account for the phenomena such as powered dynamics of group discussion (e.g., how jurors negotiated turn-taking or monopolised conversation). We also did not delve deeply into the ways that participants might have co-constructed meaning differently across the juries. Such work has been undertaken in other disciplines (e.g., Maynard and Manzo, 1993), which are better suited to situate jury deliberation utterances in a dynamic context.

Finally, it is worth acknowledging that conceptualizations of punishment throughout these studies are based on Western, individualistic cultural values. Specifically, the POQ (Yamamoto and Maeder, 2019) does not feature a range of other punitive goals. Indigenous Peoples, for instance, might prefer a restorative justice approach to dealing with criminal offences, in which the community works together to prioritise healing (Achtenberg, 2015).

### Conclusion

What was in the black box? Results demonstrate that ideas about appropriate punishment feature in both thought process and persuasion, and that mock jurors' decisions stem partially from moral conceptualizations of insanity rather than the evidence alone. Findings also provide a glimpse into the power of mixed methodology and highlight limitations of the quantitative paradigm in providing a complete understanding of narratives in unique discursive contexts.

## Data Availability Statement

The datasets presented in this study can be found in online repositories. The names of the repository/repositories and accession number(s) can be found at: https://osf.io/bwr3g/?view_only=88e67659f134493c82e4c0a928f17865.

## Ethics Statement

The studies involving human participants were reviewed and approved by Carleton University Research Ethics Board B. The patients/participants provided their written informed consent to participate in this study.

## Author Contributions

SY and EM contributed to conception/design of the study and creation of stimuli. SY conducted data collection and analyses under the supervision of EM. SY wrote the first draught of the manuscript. EM revised, read, and approved the submitted manuscript. Both authors contributed to the article and approved the submitted version.

## Conflict of Interest

The authors declare that the research was conducted in the absence of any commercial or financial relationships that could be construed as a potential conflict of interest.

## Footnotes

^1^It is important to acknowledge the ongoing debate about appropriate terminology when discussing this topic. Some argue that terms such as “disorder” and “illness” pathologize what would be better termed “mental health conditions.” Others prefer that these conditions are recognized in the same way as other physical illnesses. Throughout this paper, we mirror both the DSM and the law in using the term “disorder”.

^2^The POQ and IDA-R were counterbalanced, but there were no significant differences in reported analyses on the basis of presentation order.

^3^https://osf.io/bwr3g/?view_only=88e67659f134493c82e4c0a928f17865.

^4^Due to the correlations between POQ and IDA-R scores, as well as between the POQ dimensions, we included only a combined punishment orientation score as a predictor.

^5^We examined data on all five moral foundations, but the purity and ingroup dimensions only yielded a handful (48 and 55, respectively) of usages, most of which were contextually invalid. Therefore, we only analyzed fairness, harm, and authority. Implications are explored in the Discussion section.
